# Correction: Synthesis and preclinical application of a Prussian blue-based dual fluorescent and magnetic contrast agent (CA)

**DOI:** 10.1371/journal.pone.0295460

**Published:** 2023-11-30

**Authors:** Nikolett Hegedűs, László Forgách, Bálint Kiss, Zoltán Varga, Bálint Jezsó, Ildikó Horváth, Noémi Kovács, Polett Hajdrik, Parasuraman Padmanabhan, Balázs Gulyás, Krisztián Szigeti, Domokos Máthé

The last two authors, Krisztián Szigeti and Domokos Máthé, should be noted as contributing equally to this work.
