# Supplementary material for: Synthesis and preclinical application of a Prussian blue-based dual fluorescent and magnetic contrast agent (CA)
Source: PLoS One. 2022 Jul 20;17(7):e0264554. doi: 10.1371/journal.pone.0264554 (PMC9299340; doi:10.1371/journal.pone.0264554)
Supplement: S1 File — (DOCX) [file pone.0264554.s001.docx]

Results and discussion

Synthesis of uncoated Prussian Blue nanoparticles and Prussian Blue nanoparticle complexes

Initially, we experienced excipitation of nanoparticle residues during the synthesis, with either increase of the concentration or the increase of the temperature. We did succeed to progress towards a stable species, however we did not focus on this issue.

Herein we describe the method how we produced the stable uncoated PBNP species, using wide variety of methods such as syrange filters, differential centrifugation and ion exchange resins. The reaction solutions were separated via differential velocity centrifugation, where the initial reaction solutions of PB-HCl were differentiated using different centrifugal forces. In case of Sample D, we used 1000 x g for 10 minutes, 2 times, and 2000 x g for 10 minutes 2 times. Then the solutions were measured via dynamic light scattering. The results of the DLS measurements are show non Figure S1.


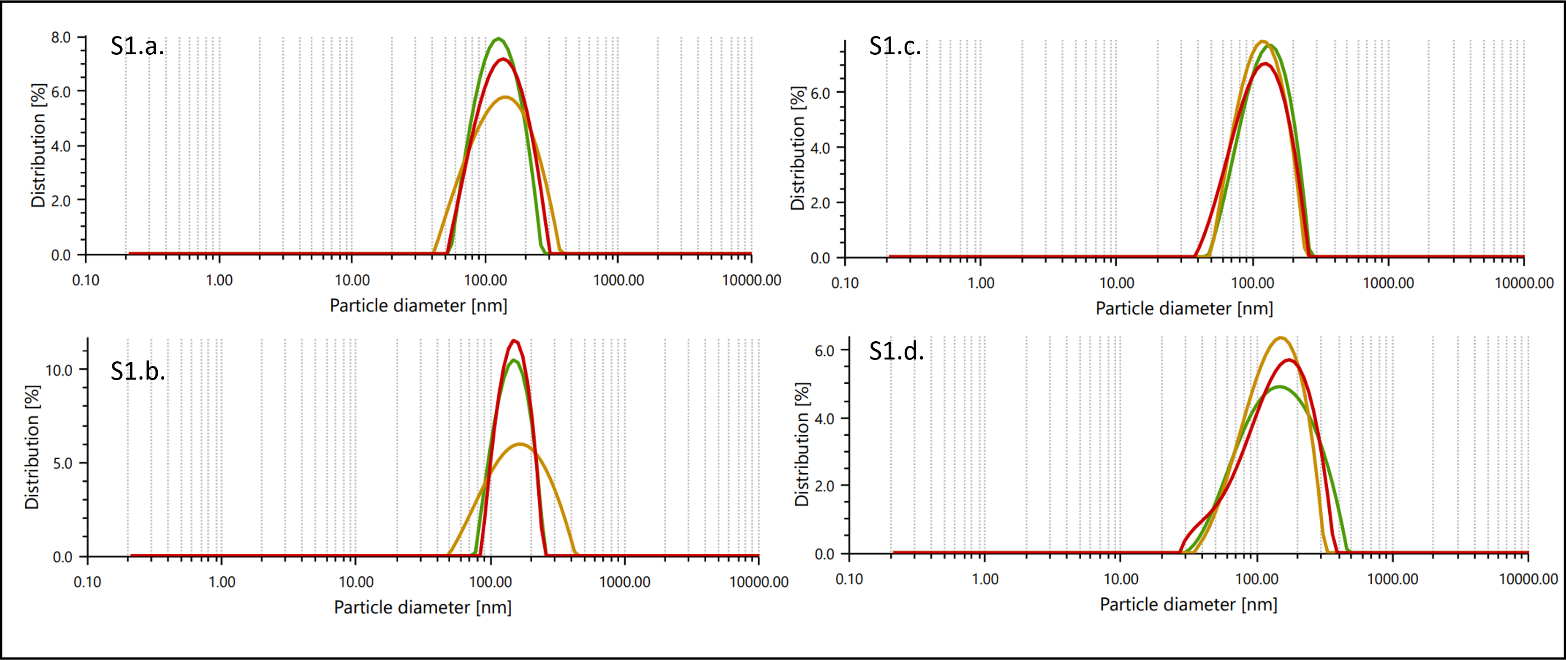
Samples E, F and G represent the PNBP-complex results, differentiated via centrifugation using 1000 x g for 10 minutes 1 or 2 times (Sample E and F) or using 0.22 µm membrane filter and cetrifugation of the sample with 2000 x g for 10 minutes respectively (Sample G)

Figure S1 describes the effect of differential velocity centrifugation of the reaction solution of PB-HCl. S1.a. Shows the effect of low g differential velocity centrifucation on the sedimentation of PB-HCl reaction solutions (Sample A; 1000 x g for 10 minutes). S1.b. The effect of low g differential centrifugation on the PB-HCl reaction solutions (Sample B; 2 times 1000 x g for 10 minutes). S1.c. The effect of repeated low g differential centrifugation (Sample C; 2 times 1000 x g for 10 minutes and 1 time 2000 x g for 10 minutes). S1.d. The effect of repeated low g differential centrifugation (Sample C; 2 times 1000 x g for 10 minutes and 2 times 2000 x g for 10 minutes).


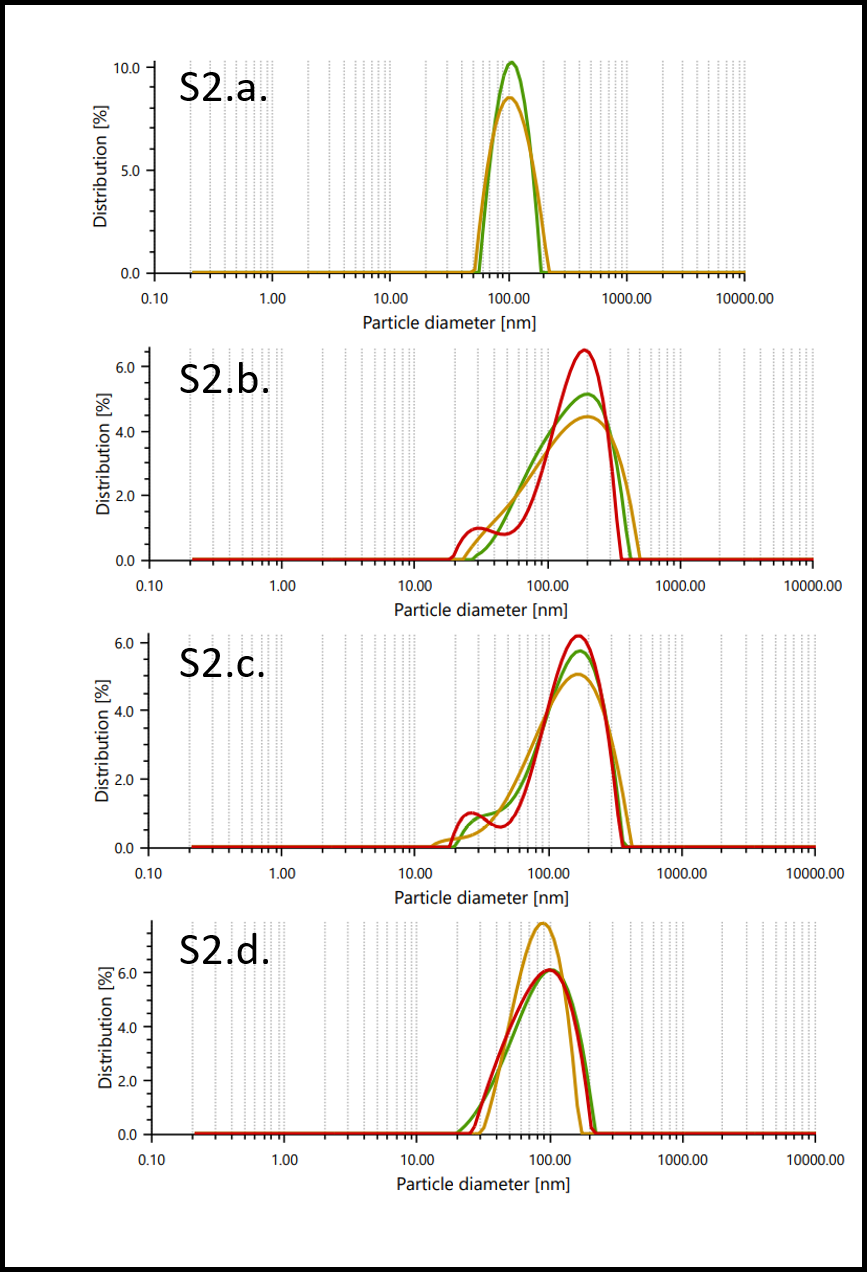
Figure S2 shows the DLS measurement in the nanoparticle solutions. Fig. S2.a. Shows the results of Sample D (2 times 1000 x g for 10 minutes plus 2 times 2000 x g for 10 minutes), followed by the final centrifugation on 21130 x g for 30 minutes. Fig. S2.b. S2.c. and S2.d. show the result of Samples E, F and G respectively.

Figure S3 shows the TEM measurement of the nanoparticle solutions. Fig. S3.a. and S3b. show the results of Sample F (2 times 1000 x g for 10 minutes), followed by the final centrifugation on 21130 x g for 30 minutes. Fig. S3.c. and S2.d. show the results of Samples G
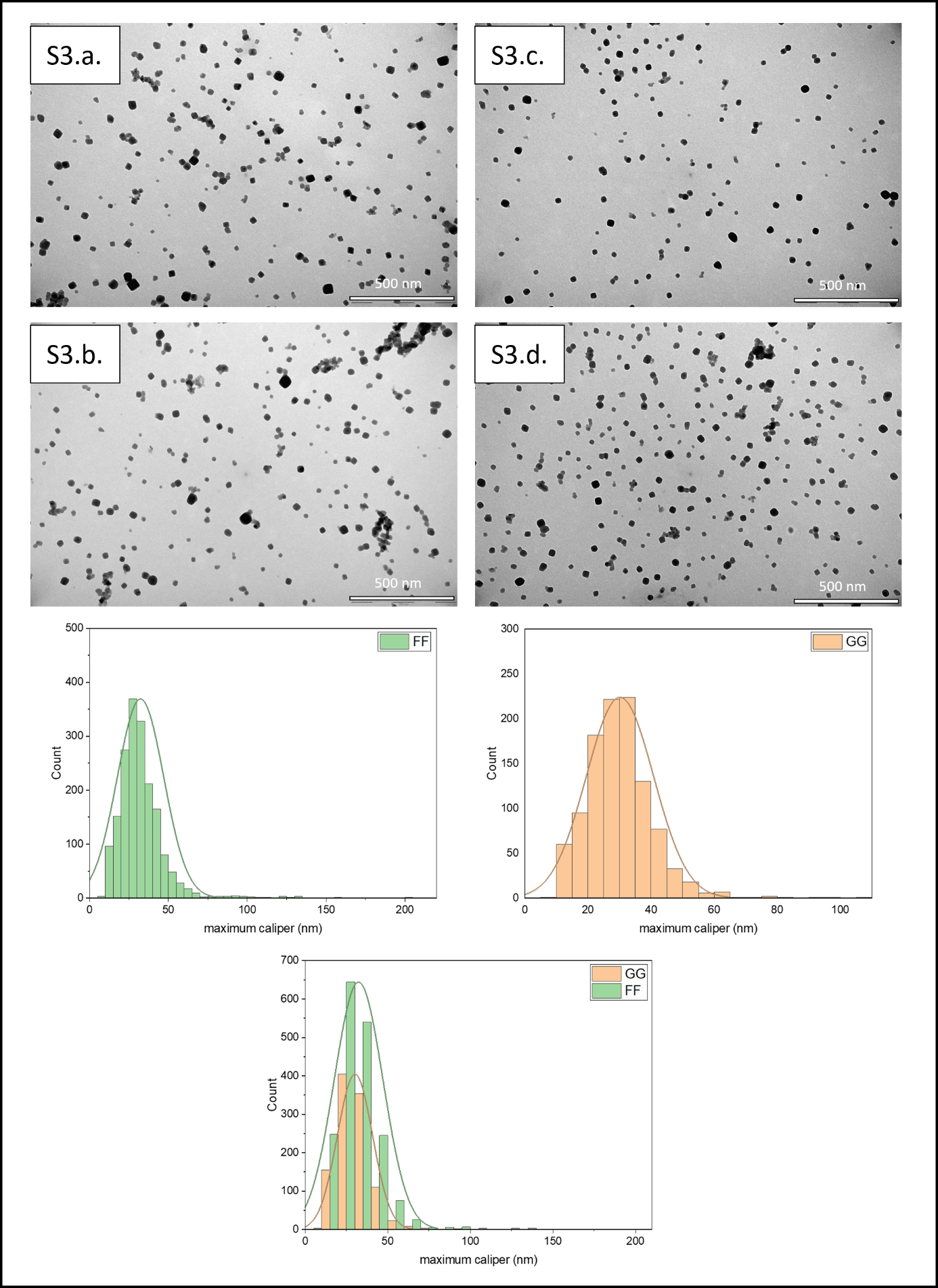
 (Filtered through a 0.22 µm membrane and centrifuged 1 time 2000 x g for 10 minutes). The histograms of Figure S3 represent the size-distributions of the nanoparticle solutions respectively.


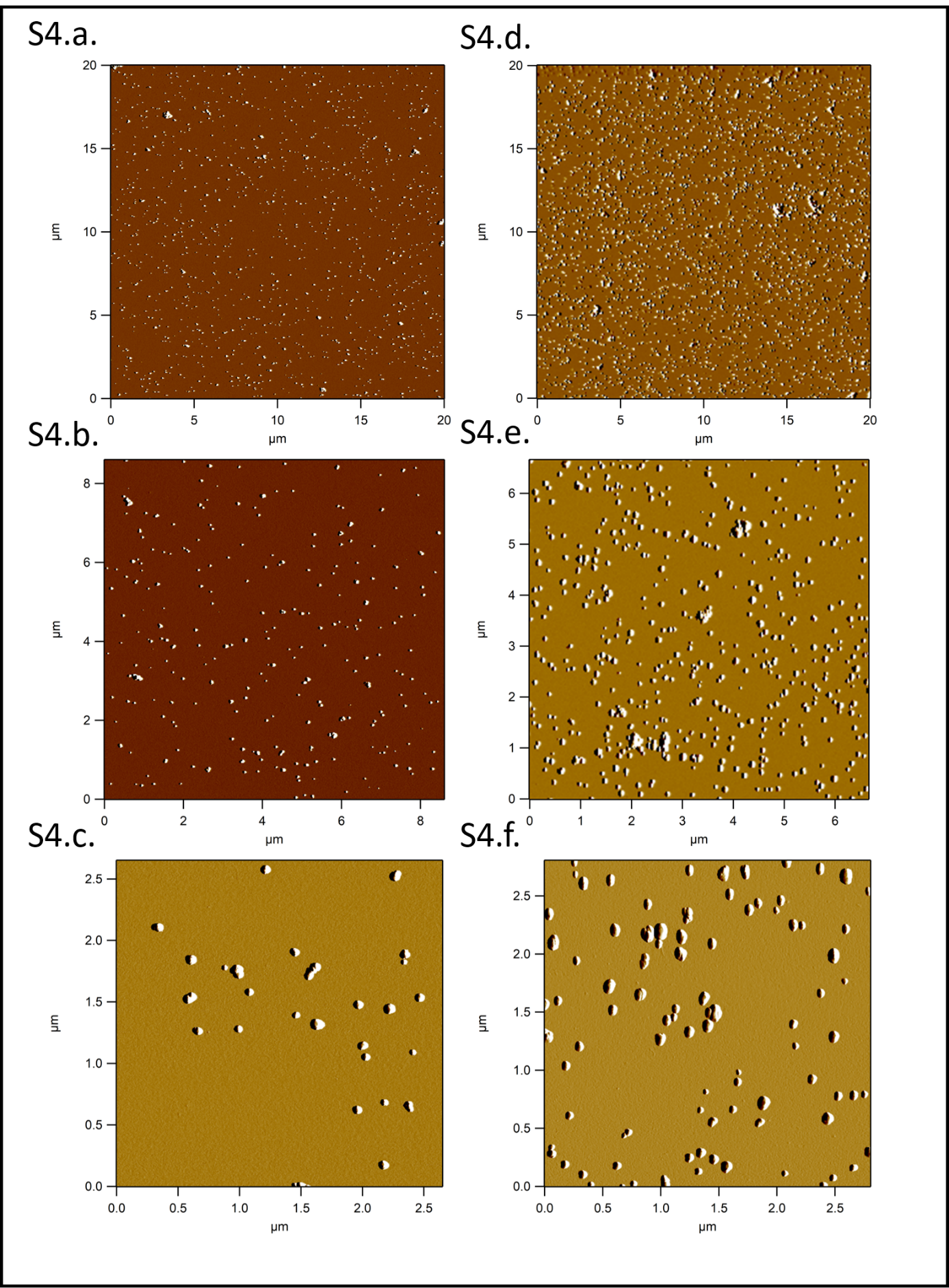


Figure S4 shows the AFM measurement of the nanoparticle solutions. Fig. S4.a., S4b. and S4.c. show the results of Sample F (2 times 1000 x g for 10 minutes), followed by the final centrifugation on 21130 x g for 30 minutes. Fig. S4.d., S4.e. and S4.f. show the results of Samples G (Filtered through a 0.22 µm membrane and centrifuged 1 time 2000 x g for 10 minutes).
