# Supplementary material for: Synthesis and preclinical application of a Prussian blue-based dual fluorescent and magnetic contrast agent (CA)
Source: PLoS One. 2022 Jul 20;17(7):e0264554. doi: 10.1371/journal.pone.0264554 (PMC9299340; doi:10.1371/journal.pone.0264554)
Supplement: Attachment — Submitted filename: PONE-D-21-26225.docx [file pone.0264554.s002.docx]

Synthesis and preclinical application of a Prussian blue-based dual fluorescent and magnetic contrast agent (CA), I appreciate the authors for the contribution for this manuscript.

However, the manuscript needs major revision in the below mentioned sections.

Abstract: Authors need to mention the brief summary of results in few lines

Authors published one recent article on “Fluorescent, Prussian Blue-Based Biocompatible Nanoparticle System for Multimodal Imaging Contrast” What is the difference and novelty of the current manuscript as compared to published one. Method for the preparation is more or less similar except temperature.

Why there is an elevated temperature used in current method like 60°C as compared to published method 40°C?

In results authors should mention the DLS graphs for representation of particle size distribution

Discussion of the results is not adequate; Authors should elaborate the discussion section in the manuscript

Figure:1 Clarity is missing, authors should change the figure

Figure 2 b: By looking at the TEM image the size of the particles are less than 100 nm which is 49 nm, but the authors mentioned size of complex is 120.41 ± 14.99 nm, kindly provide the TEM and DLS image for the complex as well.

Authors might have provided the toxicity studies of the synthesized complex/CA : in cell line and animal model

Minor corrections

Grammatical errors need to be resolved in the manuscript

Like surface area : nm^2^, super and subscripts as well as ./, in the manuscript.
